# Diverse Banana Pseudostems and Rachis Are Distinctive for Edible Carbohydrates and Lignocellulose Saccharification towards High Bioethanol Production under Chemical and Liquid Hot Water Pretreatments

**DOI:** 10.3390/molecules26133870

**Published:** 2021-06-24

**Authors:** Jingyang Li, Fei Liu, Hua Yu, Yuqi Li, Shiguang Zhou, Yuanhang Ai, Xinyu Zhou, Youmei Wang, Lingqiang Wang, Liangcai Peng, Yanting Wang

**Affiliations:** 1 Biomass & Bioenergy Research Centre, College of Plant Science & Technology, Huazhong Agricultural University, Wuhan 430070, China; jingyanglee@163.com (J.L.); feiliu2021@foxmail.com (F.L.); yh201906@foxmail.com (H.Y.); pengpeng2danshuang@163.com (S.Z.); yuanhangai@webmail.hzau.edu.cn (Y.A.); zhou1997student@163.com (X.Z.); wym_quiet@yeah.net (Y.W.); lqwang@mail.hzau.edu.cn (L.W.); lpeng@mail.hzau.edu.cn (L.P.); 2 Laboratory of Biomass Engineering & Nanomaterial Application in Automobiles, College of Food Science & Chemical Engineering, Hubei University of Arts and Science, Xiangyang 441053, China; yuqi.li@hotmail.com; 3 Haikou Experimental Station, Chinese Academy of Tropical Agricultural Sciences, Haikou 570102, China; 4 State Key Laboratory for Conservation & Utilization of Subtropical Agro-Bioresources, College of Agriculture, Guangxi University, Nanning 530000, China

**Keywords:** banana, pseudostem, rachis, biomass pretreatments, enzymatic saccharification, bioethanol fermentation, cellulose crystallinity

## Abstract

Banana is a major fruit crop throughout the world with abundant lignocellulose in the pseudostem and rachis residues for biofuel production. In this study, we collected a total of 11 pseudostems and rachis samples that were originally derived from different genetic types and ecological locations of banana crops and then examined largely varied edible carbohydrates (soluble sugars, starch) and lignocellulose compositions. By performing chemical (H_2_SO_4_, NaOH) and liquid hot water (LHW) pretreatments, we also found a remarkable variation in biomass enzymatic saccharification and bioethanol production among all banana samples examined. Consequently, this study identified a desirable banana (Refen1, subgroup Pisang Awak) crop containing large amounts of edible carbohydrates and completely digestible lignocellulose, which could be combined to achieve the highest bioethanol yields of 31–38% (% dry matter), compared with previously reported ones in other bioenergy crops. Chemical analysis further indicated that the cellulose CrI and lignin G-monomer should be two major recalcitrant factors affecting biomass enzymatic saccharification in banana pseudostems and rachis. Therefore, this study not only examined rich edible carbohydrates for food in the banana pseudostems but also detected digestible lignocellulose for bioethanol production in rachis tissue, providing a strategy applicable for genetic breeding and biomass processing in banana crops.

## 1. Introduction

Banana is one of the most consumed fruits in the world. However, it not only provides a high yield of edible carbohydrates with high nutrition but also produces large amounts of pseudostem and rachis residues rich in lignocellulose [1]. Hence, various genetic types of banana plants have been grown in different ecological regions for diverse banana fruits and lignocellulose residues [2]. Although biomass process technology has been implemented for banana pseudostem utilization, it is important to explore efficient biomass enzymatic saccharification for high bioethanol production among the different types of banana crops, particularly for the banana rachis residues [3,4]. In addition, much remains unknown about edible carbohydrate accumulation in both pseudostem and rachis residues of different banana crops [5].

Due to global warming and fossil energy overconsumption, lignocellulose is increasingly considered as a sustainable biomass resource, and cellulosic bioethanol has been evaluated as a promising solution for partial replacement of fossil fuels [6,7,8]. However, the natural recalcitrance of lignocellulose fundamentally creates inefficient biomass enzymatic saccharification for costly bioethanol conversion [9,10].

Biomass recalcitrance is in principle determined by wall polymer feature and wall network construction [11,12]. In general, cellulose crystalline index has been considered as the key negative factor affecting biomass enzymatic saccharification [13,14]. In comparison, hemicellulose could negatively affect cellulose crystallinity, and particularly its two major monosaccharides (xylose and arabinose) proportions have been recently examined as a major factor in lignocellulose enzymatic hydrolysis [15,16]. In addition, lignin is well characterized as a barrier against cellulases enzyme loading and accession, but three lignin monomers (S, G, H) proportions could play dual roles in biomass enzymatic hydrolyses, mainly due to the pretreated lignocellulose residues with distinctive structures in bioenergy crops examined [17,18].

In the past few years, various physical and chemical pretreatments have been carried out to enhance sequential enzymatic hydrolysis and final bioethanol production [19,20,21,22]. For instance, liquid hot water (LHW) has been applied as a green-like (non-chemical) pretreatment, whereas acid and alkali pretreatments are conducted for distinct wall polymer extraction and polymer feature modification [23,24]. However, most pretreatments require extreme conditions such as high chemical concentrations and incubation temperatures, which causes costly biomass processing with potential secondary waste release [25]. Nevertheless, recent reports show that the milder diluted acid/base and green-like pretreatments are sufficient for complete biomass enzymatic hydrolysis in recalcitrance-reduced lignocellulose residues by selecting desirable bioenergy plants, genetic mutants, and transgenic crops [26]. For example, LHW pretreatment is conducted for maximized bioethanol production in brittle corn stalk [24], whereas mild chemical pretreatments are sufficient for complete biomass enzymatic saccharification in transgenic rice straws [27].

In the present study, therefore, we collected a total of 11 pseudostem and rachis samples derived from eight distinct genetic types and original locations of banana crops and determined largely varied edible carbohydrates (soluble sugars, starch) contents and lignocellulose compositions. We then performed mild chemical (acid, alkali) and LHW pretreatments to compare biomass enzymatic saccharification and bioethanol production among all banana samples examined. This study also detected major wall polymer features to explain why the desirable banana samples were of high biomass saccharification and bioethanol production.

## 2. Results and Discussion

### 2.1. Large Variations in Edible Carbohydrates and Lignocelluloses among Different Genetic Types of Banana Crops

**Figure 1:**
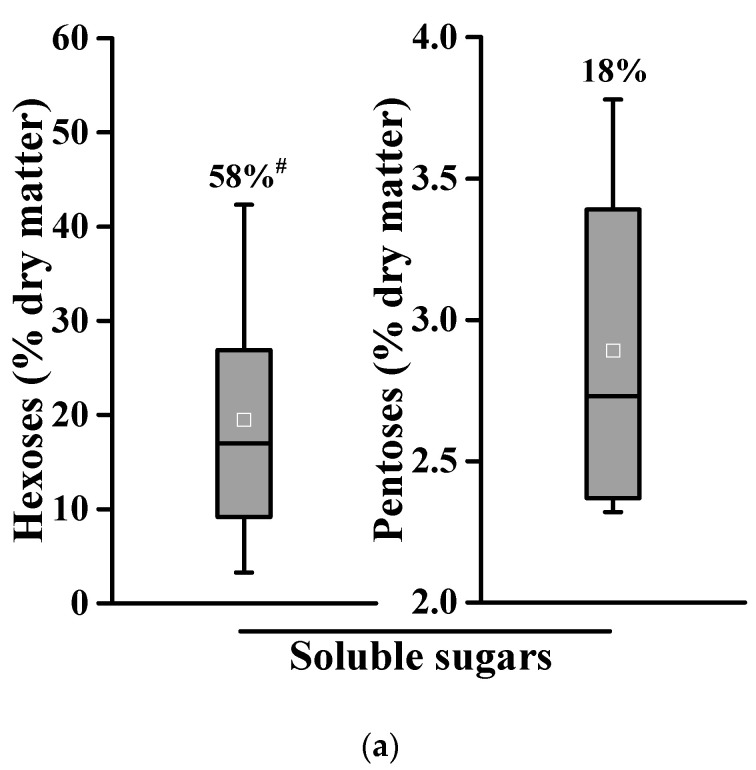

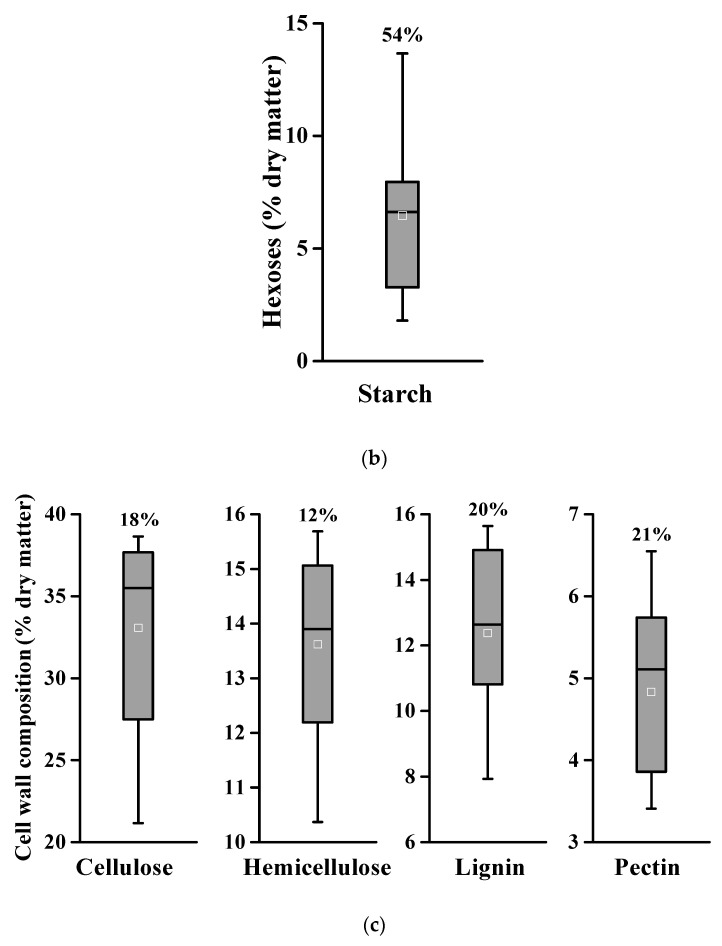
Variation of edible carbohydrates and lignocellulose compositions among 11 total banana samples. (**a**) Soluble sugars, (**b**) Starch, (**c**) Wall polymer composition. ^#^ As coefficient of variation/CV by calculating SD value divided by mean (*n* = 11).

As banana is a fruit plant grown worldwide in different ecological regions, this study collected nine pseudostems and two rachis samples from Haikou experimental fields, which are originally derived from six countries with eight distinct genetic types (Table S1). Among the 11 total banana samples, this study initially determined soluble sugar contents including hexoses ranged from 3.3% to 42.3% (% dry matter) and pentoses from 2.3% to 3.8% with a coefficient of variation (CV) of 58% and 18%, respectively (Figure 1a; Table 1).

Meanwhile, the 11 total banana samples showed much-varied starch levels from 1.8% to 13.7% with CV at 54% (Figure 1b). In particular, the pseudostem (PS-2) sample of the Refen1 banana contains the highest starch level and the second-highest soluble hexoses content, whereas its rachis sample (RC-2) covered the highest soluble hexoses level among all banana samples examined (Table S1), suggesting that the Refen1 banana crop (PS-2, RC-2) could be applied to extract large amounts of edible carbohydrates for food purposes.

Furthermore, we examined a remarkably varied wall polymer composition such as 21.2–38.7% cellulose (% dry matter), 10.4–15.7% hemicellulose, 7.9–15.7% lignin, and 3.4–6.6% pectin with CV values of 18%, 12%, 20%, and 21%, respectively (Figure 1c). Hence, among the different types of banana crops, we could extract large amounts of edible carbohydrates, but were also able to identify digestible lignocellulose substrates in the desirable pseudostems and rachis samples as described below.

### 2.2. Acid Pretreatment for Varied Biomass Enzymatic Saccharification among Banana Lignocellulose Samples

**Figure 2:**
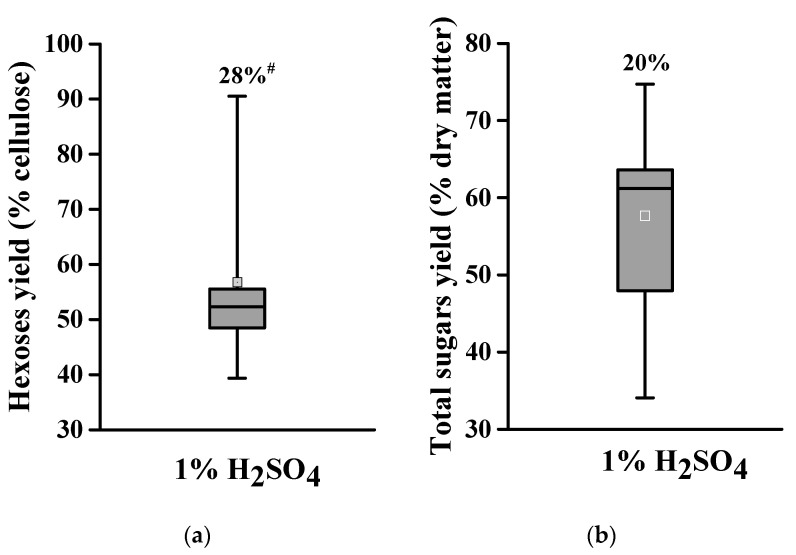
Variation of biomass enzymatic saccharification among 11 total banana samples after acid pretreatments (*n* = 11). (**a**) Hexoses yields released from enzymatic hydrolysis after 1% H_2_SO_4_ pretreatment; (**b**) total sugar yields (hexoses and pentoses) released from both enzymatic hydrolysis and 1% H_2_SO_4_ pretreatment; ^#^ As coefficient of variation.

With respect to the banana pseudostems and rachis that were of diverse cell wall compositions as described above, this study determined their biomass enzymatic saccharification by measuring either hexoses yield (% cellulose) released from enzymatic hydrolysis of pretreated lignocellulose or total sugars (hexoses and pentoses) yield (% dry matter) obtained from both enzymatic hydrolysis and pretreatment. Using our previously established condition, this work performed acid pretreatments at low concentrations (1% H_2_SO_4_) and measured the hexoses yields, which ranged from 14.4% to 21.1% (% cellulose), and total sugars yields, which ranged from 19.3% to 58.3% (% dry matter) with CV at 28% and 20% among the 11 total banana samples (Figure 2).

In particular, the pseudostems (PS-2) and rachis (RC-2) samples of Refen1 banana crop showed much higher total sugar yields (58.3%, 51.2%) than those of all other banana samples (19.3–43.2%), suggesting that this banana crop should be of the desirable lignocellulose substrate for biomass enzymatic saccharification under acid pretreatment. However, compared to other grassy crops examined in the previous studies [24,25,26,27,28,29,30], both banana pseudostems and rachis residues showed relatively low enzymatic saccharification from the 1%H_2_SO_4_ pretreatments performed in this study.

### 2.3. Alkali Pretreatments for Much Enhanced Biomass Enzymatic Saccharification

**Figure 3:**
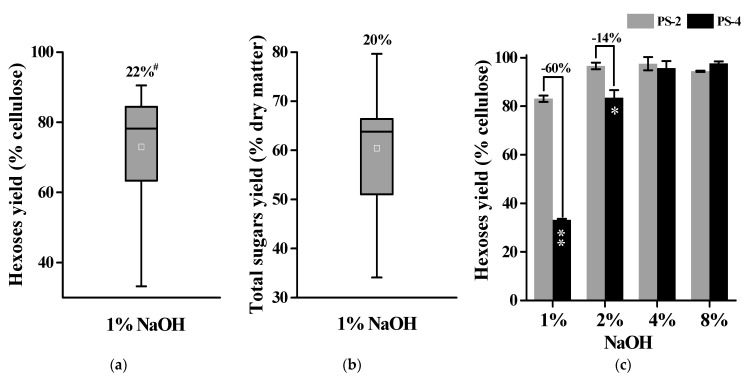
Variation of biomass enzymatic saccharification among 11 total banana samples after alkali pretreatments (*n* = 11). (**a**) Hexoses yields released from enzymatic hydrolysis after 1% NaOH pretreatment; (**b**) total sugar yields (hexoses and pentoses) released from both enzymatic hydrolysis and 1% NaOH pretreatment; (**c**) hexoses yields released from enzymatic hydrolysis after four concentrations of NaOH pretreatments with two banana samples; ^#^ As coefficient of variation. * and ** indicate significant differences between PS-2 and PS-4 by *t*-test at *p* < 0.05 and *p* < 0.01 (*n* ± 3). Minus sign percentage was calculated by subtraction between PS-2 and PS-4 values divided by PS-2. Data as mean SD (*n* ± 3).

Since the acid pretreatment caused relatively low enzymatic saccharification, this study performed alkali pretreatment with all 11 banana samples. As a result, the banana samples showed largely varied biomass enzymatic saccharification with the hexoses yields ranging from 12.6% to 30.52% (% cellulose) and total sugars yields ranging from 20.4% to 65.2% (% dry matter) with CV at 22% and 20% after 1% NaOH pretreatment with the mild (50 °C for 1 h) condition (Figure 3a,b).

Furthermore, this study performed pretreatments with high concentrations of alkali (2%, 4%, 8%NaOH) in two representative banana pseudostems samples (PS-2, PS-4), which respectively represent the highest and lowest hexoses yields from the 1% NaOH pretreatment performed above. Under 4% NaOH pretreatments, two banana samples showed the hexoses yields of 96% and 98% for almost complete biomass enzymatic saccharification (Figure 3c). Hence, the alkali pretreatment should be effective for sequential biomass enzymatic saccharification in banana lignocellulose residues.

### 2.4. Liquid Hot Water Pretreatments for Complete Biomass Saccharification in Banana Rachis Samples

**Figure 4:**
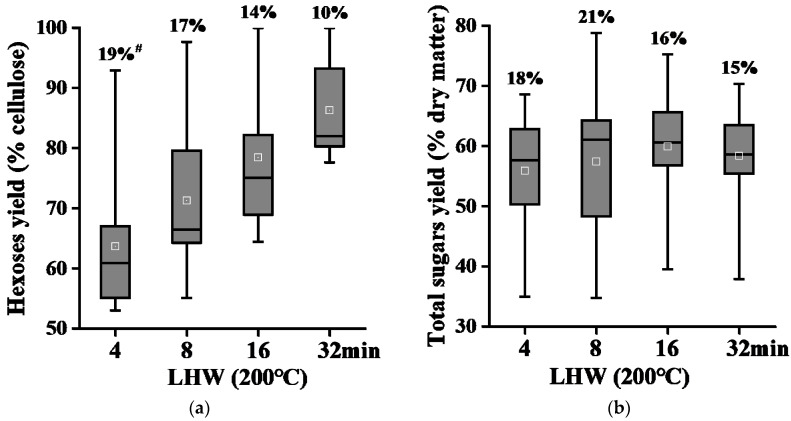
Variation of biomass enzymatic saccharification among 11 total banana samples after liquid hot water (LHW) pretreatments (*n* = 11). (**a**) Hexoses yields released from enzymatic hydrolysis after a time course of LHW pretreatments; (**b**) total sugar yields (hexoses and pentoses) released from both enzymatic hydrolysis and a time course of LHW pretreatments; ^#^ As coefficient of variation.

Provided that the alkali pretreatment at high concentration could cause almost complete biomass enzymatic saccharification in the banana samples examined above, it remains to recycle all chemicals and other wastes. In this study, we further performed green-like (non-chemical) LHW pretreatments with all banana samples at a time course (4, 8, 16, 32 min) using our previously established condition (Figure 4).

Under the 4 min LHW pretreatment, all banana samples showed the hexoses yields at more than 50% (% cellulose) with CV at 19% (Figure 4a). Notably, under 16 min LHW pretreatment, two rachis samples had a complete biomass enzymatic saccharification with hexoses yields at 96.2% and 100%, but the pseudostems samples had hexoses yields ranging from 64.4% to 82.2%. Even though under 32 min LHW pretreatment, only two pseudostems samples showed the hexoses yields at more than 90%, other samples had hexoses yields ranging from 77.7% to 84.5%. Meanwhile, under the time course of LHW pretreatments, all banana samples had largely varying total sugar yields (% dry matter), with CV at 18%, 21%, 16%, and 15% (Figure 4b). Hence, the LHW pretreatment should be specifically effective for biomass enzymatic saccharification of banana rachis tissues.

### 2.5. Remarkably High Bioethanol Production in the Desirable Banana Samples

**Figure 5:**
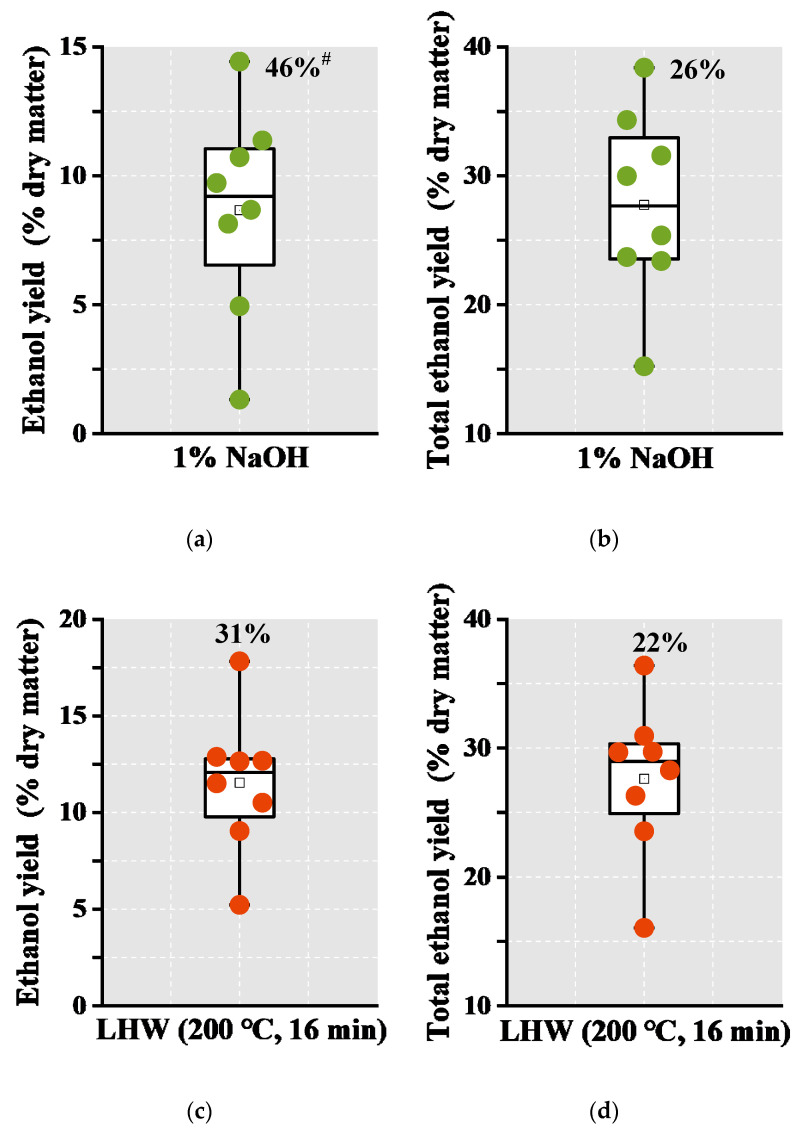
Variation of bioethanol production obtained from yeast fermentation among 8 total banana samples (*n* = 8). (**a**) Bioethanol yields using total hexoses released from enzymatic hydrolysis after 1% NaOH pretreatments; (**b**) Estimated total bioethanol yields using all hexoses and pentoses from soluble sugars, starch, and enzymatic hydrolysis after 1% NaOH pretreatments; (**c**) bioethanol yields using total hexoses released from enzymatic hydrolysis after 16 min LHW pretreatments; (**d**) estimated total bioethanol yields using all hexoses and pentoses from soluble sugars, starch, and enzymatic hydrolysis after 16 min LHW pretreatments; ^#^ As coefficient of variation.

Using total hexoses obtained from enzymatic saccharification, this study conducted a classic yeast fermentation to achieve bioethanol production from eight representative banana samples (Figure 5; Table S2).

Their results showed bioethanol yields of the banana samples from 1.3% to 14.4% (% dry matter), with CV at 46%, by using hexoses released from enzymatic hydrolysis upon 1% NaOH pretreatment (Figure 5a), which is consistent with their varied biomass enzymatic saccharification. Based on the average xylose-ethanol conversion rate of 35% and glucose-ethanol rate of 51% as previously reported [31,32], this study further evaluated total bioethanol yields, ranging from 15.2% to 38.4% (% dry matter) with CV at 26%, by using all hexoses and pentoses from soluble sugars, starch, and enzymatic hydrolysis of alkali pretreated biomass residues (Figure 5b). Meanwhile, under 16 min LHW pretreatment, the banana samples also had bioethanol yields from 5.2% to 17.8% (% dry matter) and total ethanol yields from 16% to 36.4% (Figure 5c,d). Hence, due to its highest levels of soluble sugars and starch and almost complete lignocellulose saccharification among the banana samples examined, the banana (Refen1) crop could be selected to achieve the highest bioethanol yields in both pseudostems (PS-2) and rachis (RC-2) samples (Table 2), compared with the previously reported ones in other bioenergy crops. On the other hand, even though the PS-2 sample showed relatively lower lignocellulose enzymatic saccharification than that of the RC-2 sample, the PS-2 sample contained much more soluble sugars and starch for relatively higher total bioethanol yield. It thus suggested that the banana (Refen1) cultivar could not only provide large amounts of edible carbohydrates (soluble sugars, starch) for food as an excellent fruit crop, but it may also be applied as the desirable bioenergy crop for high bioethanol production.

### 2.6. Characteristic Lignocellulose Features for Distinct Biomass Enzymatic Saccharification in the Desirable Banana Sample

**Figure 6:**
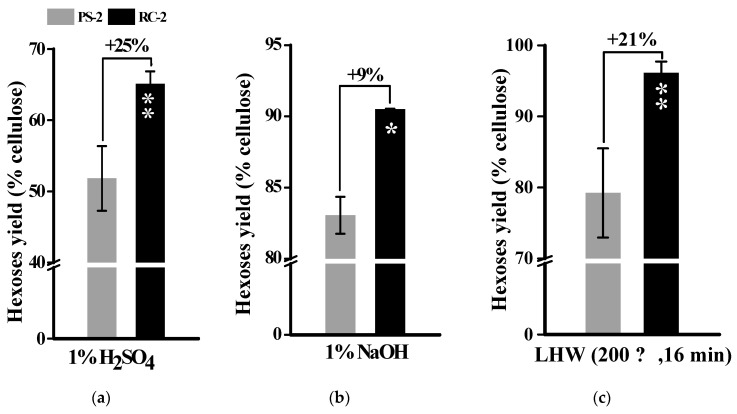
Comparison of hexoses yields (% cellulose) released from enzymatic hydrolysis after chemical and LHW pretreatments between PS-2 and RC-2 samples; (**a**) 1% H_2_SO_4_ pretreatment; (**b**) 1% NaOH pretreatment; (**c**) LHW pretreatment; * and ** As significant differences between the RC-2and PS-2 by Student’s *t*-test at *p* < 0.05 and 0.01 (*n* = 3) with the increased (+) percentage of the RC-2 sample relative to the PS-2; Data as means ± SD (*n* = 3).

Because the pseudostems and rachis residues of banana crops showed distinct biomass enzymatic saccharification under chemical and LHW pretreatments (Figure 6), this study examined major wall polymer proportions and features in the desirable banana (Refen1) cultivar. In general, both pseudostems (PS-2) and rachis residues (RC-2) of the Refen1 cultivar showed high cellulose levels at 52% and 51% (of total wall polymers) and pectin contents at 11% and 7.9%, with similar hemicellulose levels of 22% and 23% (Table 3), confirming that the banana (Refen1) cultivar, rich in polysaccharides, could be applied as a desirable bioenergy crop.

However, as the lignin deposition contributes to lignocellulose recalcitrance against biomass enzymatic saccharification [37,38,39], this study detected that the rachis residue (RC-2) had significantly higher lignin levels than the pseudostems (PS-2) by 31%, suggesting that the wall polymer features of the RC-2 sample, rather than its lignin level, may predominately affect biomass enzymatic hydrolysis after chemical (acid, alkali) and LHW pretreatments performed in this work (Figure 6).

**Figure 7:**
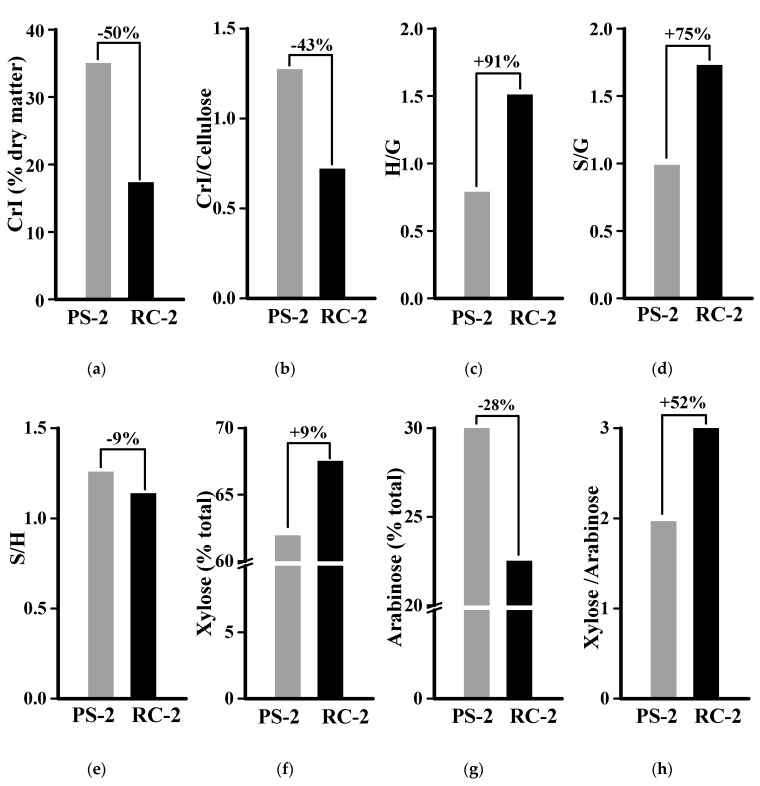
Comparison of major wall polymer features between PS-2 and RC-2 samples. (**a**) Cellulose CrI against dry matter; (**b**) CrI against cellulose level; (**c**–**e**) three lignin monomer ratios; (**f**,**g**) xylose and arabinose levels of hemicellulose; (**h**) xylose/arabinose ratios. ± was calculated by subtraction between PS-2 and PS-4 values divided by PS-2. Data as mean SD (*n* ± 3).

To test this assumption, this study examined cellulose crystallinity, which has been considered as the key negative factor on lignocellulose’s recalcitrant property [13,14]. Compared to the PS-2 sample, the RC-2 residue showed significantly reduced cellulose crystallinity by 50% and 43% on the basis of crystalline index (CrI) values against dry matter and cellulose (Figure 7a,b), which was consistent with its higher biomass enzymatic saccharification (Figure 6). In terms of three lignin monomer (H, S, G) proportions, this study showed that the RC-2 had remarkably higher H/G and S/G ratios than those of the PS-2 with small different S/H ratios (Figure 7c–e). As G-monomer of lignin could play a major role in lignin interaction with wall polysaccharides [40,41], the data suggest that relatively low G proportion of the RC-2 residue may be a major lignin factor accounting for its high biomass enzymatic saccharification relative to the PS-2 residue. It was also consistent with the recent findings that three lignin monomers are of dual impact on biomass enzymatic saccharification of different lignocellulose substrates [28,42]. In addition, compared to the PS-2 sample, the RC-2 residue was of relatively higher xylose and lower arabinose levels, leading to much raised xylose/arabinose ratio (Figure 7f–h). As the xylose/arabinose ratio of hemicelluloses has been characterized as a negative factor on biomass enzymatic saccharification in grassy crops examined [41,43,44], the result suggested that the hemicellulose feature may play a small role in biomass saccharification in the banana lignocellulose substrate, but it should be further explored in future study.

## 3. Material and Methods

### 3.1. Banana Samples Collection

A total of nine distinct banana crops were grown in the Fruit Experimental Field of Haikou Experimental Station, Chinese Academy of Tropical Agricultural Sciences (CATAS) in Danzhou city, Hainan province, China. After banana fruits were harvested, the remaining pseudostem and rachis residues were chopped and dried at 60 °C until constant weight. The dried biomass samples were ground into powders, passed through a 40-mesh screen, and stored in a dry container until use.

### 3.2. Plant Cell Wall Fractionation

The cell wall fractionation of the banana sample was conducted as previously described [45]. Using potassium phosphate buffer (pH 7.0), chloroform–methanol (1:1, *v*/*v*), DMSO–water (9:1, *v*/*v*), and ammonium oxalate 0.5% (*w*/*v*), the soluble sugars, lipid, starch, and pectin of biomass samples were sequentially removed. The remaining residue was then extracted with 4 M KOH containing 1.0 mg mL^−1^ sodium borohydride for the KOH-extractable hemicelluloses. The final pellet was dissolved with H_2_SO_4_ (67%, *v*/*v*) to determine cellulose and non-KOH-extractable hemicelluloses levels. All experimental analyses were completed as independent biological triplicate.

### 3.3. Colorimetric Assay of Hexoses and Pentoses and Uronic Acids

UV–VIS Spectrometer (V-1100D, Shanghai MAPADA Instruments Co., Ltd., Shanghai, China) was applied for hexoses, pentoses, and uronic acids assay as previously described [46]. Hexoses and pentoses were, respectively, detected by anthrone/H_2_SO_4_ [47] and orcinol/HCl [48] methods. Regarding the pentose interference on the hexose readings at 620 nm, the pentose deduction was completed at 660 nm with a calibration curve established to correct hexose values. Total uronic acids were assayed by *m*-hydroxybiphenyl/NaOH method [47]. For starch and cellulose assay, total hexoses were calculated by the anthrone/H_2_SO_4_ method. The hemicelluloses were calculated by determining the total hexoses and pentoses of the hemicelluloses fraction. The hexoses, pentoses, and uronic acids of the pectin fraction were calculated as total pectin. All experimental analyses were completed in independent triplicate.

### 3.4. Total Lignin and Monolignol Detection

A two-step acid hydrolysis method was used for total lignin assay, according to the Laboratory Analytical Procedure of the National Renewable Energy Laboratory [49]. Three lignin monomers were measured by HPLC (1525, Waters Corp., MA, USA) using nitrobenzene oxidation method as previously described [28].

### 3.5. Hemicellulose Monosaccharide Determination

GC-MS (SHIMADZU GCMS-QP2010 Plus) was used for the detection of monosaccharide composition of hemicellulose as previously described [50]. Trifluoroacetic acid (TFA) and *myo*-inositol were obtained from Aladdin Reagent Inc (Shanghai, China). 1-Methylimidazole was purchased from Sigma-Aldrich Co. LLC (Shanghai, China). Acetic anhydride and acetic acid were obtained from Sinopharm Chemical Reagent Co., Ltd (Shanghai, China).

### 3.6. Soluble Sugars Extraction and Assay

The biomass sample (0.300 g) was incubated with 6 mL potassium phosphate buffer (pH 4.8) in a boiling water bath for 1 h and shaken every 10 min. After centrifugation at 3000× *g* for 5 min, the supernatant was collected, and its hexoses and pentoses were respectively detected by colorimetric assay as described above. All analyses were completed by three independent experiments.

### 3.7. Detection of Cellulose Crystalline Index

X-ray diffraction method was applied to detect cellulose crystalline index (CrI) as previously described [24] using the Rigaku-D/MAX instrument (Uitima III, Japan). Technical standard errors of the CrI method were measured at ± 0.05 ~ 0.15 using five representative samples in triplicate.

### 3.8. Biomass Pretreatments

H_2_SO_4_ pretreatment: The biomass samples (0.300 g), through a 40 mesh sieve, were loaded with 6 mL H_2_SO_4_ at 1% (*v*/*v*) concentration. The sample tubes were sealed and heated at 121 °C for 20 min in autoclave (0.15 Mpa). After heating reaction, the samples were rinsed several times with distilled H_2_O until pH was 7.0, and the remaining residues were stored for enzymatic hydrolysis.

NaOH pretreatment: The well-mixed biomass samples (0.300 g) were incubated with 6 mL NaOH at various concentrations (1%, 2%, 4%, 8% *w*/*v*) shaken under 150 rpm at 50 °C for 2 h. The pellets were washed with 10 mL distilled water 5–6 times until pH was 7.0.

Liquid hot water (LHW) pretreatment: The well-mixed biomass samples (0.300 g) with 2.4 mL of distilled water were added into well-sealed stainless steel bombs and heated at 200 °C under 15 rpm shaking for 4, 8, 16, 32 min, respectively. Then, the sealed bombs were cooled down immediately and centrifuged at 3000× *g* for 5 min.

All supernatants were combined for pentoses and hexoses assay, and the remaining pellets were used for enzymatic hydrolysis as described below. All experiments were conducted in independent triplicate.

### 3.9. Enzymatic Hydrolysis of Pretreated Biomass Residues

The remaining residues obtained from pretreatments were rinsed once more with 10 mL of mixed-cellulase reaction buffer (0.2 M acetic acid–sodium acetate, pH 4.8). The washed residues were incubated with 6 mL (2.0 g L^−1^) of mixed-cellulases (containing cellulases at 13.25 FPU g^−1^ biomass and xylanase at 8.40 U g^−1^ biomass from Imperial Jade Bio-technology Co., Ltd. Ningxia, China) and shaken under 150 rpm for 48 h at 50 °C. The samples were centrifuged at 3000× *g* for 5 min, and the supernatants were collected for pentoses and hexoses assay. All experiments were carried out in independent triplicate.

### 3.10. Yeast Fermentation and Ethanol Measurement

Yeast fermentation and ethanol measurement were conducted as previously described [24,51]. Yeast of the *Saccharomyces cerevisiae* strain (purchased from Angel Yeast Co., Ltd., Yichang, China) was suspended with 0.2 M phosphate buffer (pH 4.8) for 30 min for activation prior to use. The yeast powder was then added to the phosphate buffer to achieve a final concentration of 0.5 g L^−1^ in all fermentation tubes, and the fermentation was conducted at 37 °C for 48 h in the tubes. Ethanol was measured using the K_2_Cr_2_O_7_ method. The experiments were performed in independent triplicate.

### 3.11. Statistics

Pair-wise comparisons were performed between two measurements by Student’s *t*-test. The boxplot, histogram, and regression analysis for the best fit curve were generated using Origin 8.5 software (Microcal Software, Northampton, MA, USA). The average values were obtained from the original triplicate measurements for these analyses.

## 4. Conclusions

By collecting 11 total pseudostems and rachis samples of banana crops derived from different genetic types and ecological locations, this study found largely varied edible carbohydrates (soluble sugars, starch) and lignocellulose compositions. Under chemical (H_2_SO_4_, NaOH) and LHW pretreatments, a total of 11 banana samples also showed a diverse biomass enzymatic saccharification and bioethanol production. Notably, the desirable banana (Refen1) crop not only contained large amounts of edible carbohydrates, but also showed complete biomass enzymatic saccharification from mild alkali and LHW pretreatments, which could be integrated to achieve the highest bioethanol yields of 31–38% (% dry matter) in the pseudostems and rachis samples, compared to other bioenergy crops in the previous studies. Furthermore, this study sorted out two wall polymer features (cellulose CrI and lignin G-monomer) that may play a major role in lignocellulose enzymatic hydrolysis of banana pseudostems and rachis tissues. Hence, this work has identified a desirable banana crop that was of rich edible carbohydrates and digestible lignocellulose for bioethanol production.

## Figures and Tables

**Table 1:** Edible soluble sugars and starch and lignocellulose compositions (% dry matter) among 11 total banana samples.

Samples	Soluble Sugar		Starch	Cellulose	Hemicellulose	Lignin	Pectin
	Hexoses	Pentoses					
PS-1	26.9 ± 0.6	2.4 ± 0.1	5.2 ± 0.2	35.5 ± 0.7	11.9 ± 0.7	10.8 ± 0.7	5.9 ± 0.1
PS-2	32.3 ± 1.5	3.2 ± 0.1	13.7 ± 0.5	27.5 ± 0.5	12.2 ± 0.4	7.9 ± 0.6	5.7 ± 0.1
PS-3	9.2 ± 0.7	2.5 ± 0.1	3.3 ± 0.3	38.7 ± 2.1	15.1 ± 0.3	12.6 ± 0.3	5.1 ± 0.2
PS-4	21.3 ± 0.4	3.4 ± 0.1	6.9 ± 0.5	37.7 ± 0.9	13.9 ± 0.1	12.9 ± 0.4	3.4 ± 0.1
PS-5	22.8 ± 0.2	2.9 ± 0.1	6.1 ± 0.5	37.2 ± 0.5	15.2 ± 0.1	10.9 ± 0.1	4.3 ± 0.1
PS-6	8.5 ± 0.5	2.3 ± 0.1	7.4 ± 0.4	36.7 ± 2.4	14.2 ± 0.4	12.6 ± 0.3	3.9 ± 0.1
PS-7	17.0 ± 0.5	2.6 ± 0.2	7.9 ± 0.4	32.6 ± 0.8	13.7 ± 0.2	14.9 ± 0.5	6.5 ± 0.4
PS-8	16.4 ± 0.3	2.3 ± 0.1	10.2 ± 0.1	35.1 ± 1.9	14.7 ± 0.2	15.6 ± 0.4	5.1 ± 0.2
PS-9	14.6 ± 0.4	3.8 ± 0.1	6.6 ± 0.4	37.7 ± 2.1	15.7 ± 0.2	13.0 ± 0.1	5.5 ± 0.2
RC-2	42.3 ± 0.2	3.6 ± 0.2	1.9 ± 0.2	24.1 ± 1.7	10.4 ± 0.1	9.3 ± 0.9	3.8 ± 0.1
RC-3	3.3 ± 0.3	2.7 ± 0.2	1.8 ± 0.1	21.2 ± 2.2	12.8 ± 0.4	15.5 ± 0.2	3.9 ± 0.2

Data as means ± SD (*n* = 3).

**Table 2:** Comparison of bioethanol yields achieved in this work and in the previous studies.

Material	Pretreatment	Soluble Sugars & Starch		Enzymatic Hydrolysis		Estimated Total Ethanol Yield	Ref.
		Hexoses	Pentoses	Hexoses	Pentoses		
		(% DM)	(% DM)	(% DM)	(% DM)	(% DM)	
Banana (Refen1) Pseudostem (PS-2)	1% NaOH	42.4	5.3	22.8	9.2	38.4	This study
	LHW *, 200 °C, 16 min	40.7	10.2	21.7	2.5	36.4	
Banana (Refen1)Rachis (RC-2)	1% NaOH	36.1	4.6	21.8	8.9	34.3	
	LHW, 200 °C, 16 min	33.6	8.0	23.1	11.8	30.9	
Wheat straw	Subcritical water, 220 °C, 22 min	ND	18.4	25.9	1.8	29.6	[33]
Corn straw	LHW, 200 °C, 20 min	23.7	3.6	16.5	5.1	19.3	[24]
Sweet sorghum stalk	Supercritical carbon dioxide	ND	ND	43.6	ND	22.3	[34]
Sugarcane bagasse	Sulfite-NaOH	10.2	33.3	40	16.5	26.2	[35]
Miscanthus straw	Green liquor, 32%, 150 °C, 32 min	0.1	15.1	34.2	15.1	17.1	[20]
Poplar stem	8% NaOH	5.7	3.2	31.8	ND	10.1	[36]

* Liquid hot water, DM: Dry matter, ND: Not detected.

**Table 3:** Cell wall composition (% total) in the PS-2 and RC-2 samples.

Samples	Cellulose	Hemicellulose	Lignin		Pectin	
PS-2	51.5 ± 0.3	22.9 ± 0.3	14.9 ± 0.4		10.8 ± 0.1	
RC-2	50.7 ± 0.7	21.8 ± 0.1	19.6 ± 0.2 *	+31% ^#^	7.9 ± 0.1 *	−27%

* As significant differences between the PS-2 and RC-2 samples by *t*-test at *p* < 0.05 (*n* = 3). ^#^ Percentage calculated by subtraction between the RC-2 and PS-2 values divided by PS-2. Data as mean ± SD (*n* = 3).

## Data Availability

Not applicable.

## Supplementary Materials

The following are available online. Table S1: Information of diverse banana crops for pseudostem and rachis collection; Table S2: Table S2 Variations of bioethanol yields from yeast fermentation using hexoses released from enzymatic hydrolysis after 1% NaOH pretreatment and LHW (200 °C, 16 min) in banana samples.
