# Supplementary material for: Diverse Banana Pseudostems and Rachis Are Distinctive for Edible Carbohydrates and Lignocellulose Saccharification towards High Bioethanol Production under Chemical and Liquid Hot Water Pretreatments
Source: Molecules. 2021 Jun 24;26(13):3870. doi: 10.3390/molecules26133870 (PMC8270323; doi:10.3390/molecules26133870)
Supplement: Supplementary file 1 [file molecules-26-03870-s001.zip › molecules-1211829-supplementary.pdf]

Table S1 Information of diverse banana crops for pseudostem and rachis collection.

Samples*	Vernacular name	Genetic group	Subgroup	Origin location
PS-1	Dwarf Brazilian	ABB	N/A	Hawai'i
PS-2	Pisang Awak(Refen1)	ABB	Saba	China
PS-3	Baxi	AAA	Cavendish	Australia
PS-4	Balbisiana	balbisiana	N/A	China
PS-5	Acuminata	acuminata	Burmannica	Myanmar
PS-6	Pisang(Emperor)	AA	Inarnibal	Philippines
PS-7	Red banana	AAA	Red	China
PS-8	Thousand finger	AAB	N/A	Malaysia
PS-9	Plantain	AAB	N/A	China
RC-2	Pisang Awak(Refen1)	ABB	Saba	China
RC-3	Baxi	AAA	Cavendish	Australia

* PS: Pseudostems, RC: Rachis

Table S2 Variations of bioethanol yields from yeast fermentation using hexoses released from enzymatic hydrolysis after 1% NaOH pretreatment and LHW (200 °C, 16 min) in banana samples

Samples	1% NaOH		LHW (200 °C, 16 min)	
	Ethanol yields	Total ethanol yields [a]	Ethanol yields	Total ethanol yields
PS-1	10.72	31.57	12.64	29.70
PS-2	14.43	38.38	17.82	36.40
PS-3	4.94	25.37	9.03	26.29
PS-4	9.72	23.39	12.87	28.26
PS-5	8.68	29.98	11.50	29.66
PS-6	8.14	23.69	10.50	23.52
RC-2	11.37	34.32	12.67	30.94
RC-3	1.32	15.22	5.22	16.04

[a] Estimated total ethanol yields using all hexoses and pentoses obtained from soluble sugars, starch and lignocellulose enzymatic hydrolysis, based on the average xylose-ethanol conversation rate of 35% and glucose-ethanol conversation rate of 51% as previously reported by Rodrussamee et al. (2018) and Valinhas et al. (2018).
